# Moorean tree snail survival revisited: a multi-island genealogical perspective

**DOI:** 10.1186/1471-2148-9-204

**Published:** 2009-08-18

**Authors:** Taehwan Lee, John B Burch, Trevor Coote, Paul Pearce-Kelly, Carole Hickman, Jean-Yves Meyer, Diarmaid Ó Foighil

**Affiliations:** 1 Museum of Zoology and Department of Ecology and Evolutionary Biology, University of Michigan, 1109 Geddes Ave, Ann Arbor, MI 48109-1079, USA; 2 Partulid Global Species Management Programme, B.P. 44921 Fare Tony, Papeete, Tahiti, Polynésie Française; 3 Zoological Society of London, Regents Park, London, UK; 4 University of California, Department of Integrative Biology, 3060 VLSB #3140, Berkeley, CA 94720-3140, USA; 5 Délégation à la Recherche, Ministère de l'Education, de l'Enseignement Supérieur et de la Recherche, B.P. 20981 Papeete, Tahiti, Polynésie Française

## Abstract

**Background:**

The mass extirpation of the island of Moorea's endemic partulid tree snail fauna, following the deliberate introduction of the alien predator *Euglandina rosea*, represents one of the highest profile conservation crises of the past thirty years. All of the island's partulids were thought to be extirpated by 1987, with five species persisting in zoos, but intensive field surveys have recently detected a number of surviving wild populations. We report here a mitochondrial (mt) phylogenetic estimate of Moorean partulid wild and captive lineage survival calibrated with a reference museum collection that pre-dates the predator's introduction and that also includes a parallel dataset from the neighboring island of Tahiti.

**Results:**

Although severe winnowing of Moorea's mt lineage diversity has occurred, seven of eight (six *Partula*; two *Samoana*) partulid tip clades remain extant. The extinct mt clade occurred predominantly in the *P. suturalis *species complex and it represented a major component of Moorea's endemic partulid treespace. Extant Moorean mt clades exhibited a complex spectrum of persistence on Moorea, in captivity, and (in the form of five phylogenetically distinct sister lineages) on Tahiti. Most notably, three *Partula *taxa, bearing two multi-island mt lineages, have survived decades of *E. rosea *predation on Moorea (*P. taeniata*) and in the valleys of Tahiti (*P. hyalina *and *P. clara*). Their differential persistence was correlated with intrinsic attributes, such as taxonomy and mt lineages, rather than with their respective within-island distribution patterns.

**Conclusion:**

Conservation efforts directed toward Moorean and Tahitian partulids have typically operated within a single island frame of reference, but our discovery of robust genealogical ties among survivors on both islands implies that a multi-island perspective is required. Understanding what genetic and/or ecological factors have enabled *Partula taeniata*, *P. hyalina *and *P. clara *to differentially survive long-term direct exposure to the predator may provide important clues toward developing a viable long term conservation plan for Society Island partulid tree snails.

## Background

Oceanic islands have never been connected to continental landmasses and receive their terrestrial biotas solely through trans-oceanic dispersal and subsequent *in situ *diversification [1]. Hot spot archipelagoes, in particular, are fecund cladogenic settings, and these chronologically arrayed island chains frequently accumulate species-rich endemic radiations that are of exceptional interest to evolutionary biologists [2-4]. Because they have evolved in isolation, endemic island species often lack highly developed defensive or competitive abilities [5-7], and this renders them exceptionally vulnerable to introduced continental competitors and predators [1,8,9].

One of the most pronounced recent cases of oceanic island mass extirpation has involved the rapid extinction in the wild of the large majority of the 61 described endemic Society Islands partulid tree snails (4 *Samoana *and 57 *Partula *species) following the deliberate introduction of the alien carnivorous land snail *Euglandina rosea *[10-13]. The rationale for the introduction was a biological control program aimed at another alien mollusc, the giant African land snail, *Lissachatina fulica*, and the predator was released on Tahiti in 1974, on Moorea in 1977, and on other Society Islands in the 1980s and 1990s [14].

*Euglandina rosea*'s devastating effect on non-target Society Island endemic tree snail populations is best documented for the island of Moorea [10,15]. Within a decade, all 9 Moorean partulids (7 *Partula *and 2 *Samoana *species) were deemed extirpated, but prescient interventions led to the successful establishment of off-island captive populations for most of the island's *Partula *species in international zoos and universities [15-17]. Five endemic Moorean *Partula *species (*P. taeniata*, *P. suturalis*, *P. tohiveana*, *P. mooreana *and *P. mirabilis*) have been successfully maintained in captivity for over two decades by The Partulid Global Species Management Programme, and two species (*P. exigua *and *P. aurantia*) are extinct [13]. No captive populations exist for Society Island *Samoana *species. In 1994, an experimental reestablishment of captive-reared *P. taeniata*, *P. suturalis *and *P. tohiveana *was attempted by releasing them into a 20 × 20 m^2 ^predator-proof snail field reserve erected in a Moorean valley. However, field maintenance issues led to repeated predator incursions and the experiment was terminated in 1998 [18].

The loss of the Society Island's endemic tree snails is compounded by their scientific prominence as the subject of classic studies in zoology, population biology and evolutionary genetics for over a century [19-24]. Moorean *Partula *spp., in particular, were the focus of much of this research, and have been intensively studied by B. Clarke, J. Murray, M. Johnson and their associates over a number of decades. The resulting picture is a complicated one in which six of the seven morphologically and ecologically well-defined Moorean species collectively formed two species complexes: 1) *P. taeniata *and *P. exigua*; 2) *P. suturalis*, *P. tohiveana*, *P. mooreana *and *P. aurantia*. The seventh species, *P. mirabilis*, could hybridize with either complex [22]. Moorean *Partula *spp. showed a potential for gene flow, either directly or indirectly, among all seven taxa, resulting in a lack of concordance among morphology, molecules and degree of reproductive isolation [25-28].

All Moorean partulid species were thought to be extirpated in the wild by 1987 [15] and, until recently, it looked as if Society Island partulids would survive only in captivity [13]. However, intensive on-going field surveys have detected scattered relict populations on a number of islands, including seven on Moorea. A relict Moorean population of *Samoana attenuata *has been monitored since 1996 by C. Hickman, and this species also persists on Tahiti [14,29] and on Raiatea [30]. Six surviving populations of the Moorean endemic *Partula taeniata *have been detected since 2000. This latter species was formerly distributed throughout the island and exhibited regional variation in shell phenotype [31], allozyme profile [32] and mitochondrial (mt) genetic structure [25,27].

The primary goal of our study was to assess genealogically the remnant wild Moorean partulid populations by placing them in a phylogeographic framework. A single island phylogenetic perspective is insufficient because *Samoana attenuata *has a multi-island distribution [33] and *Partula taeniata *contains divergent mt lineages that collectively form robust taxonomically polyphyletic clades with different subsets of Moorean and Tahitian congeners [25,34]. Moorea and Tahiti are neighboring islands separated by a mere 17 km of ocean. They are the largest members of the Society Island archipelago's eastern "Windward Islands/*Iles du Vent*" sub-group and support all of the sub-group's partulid populations. Moorea is the older of the two, and allozyme and morphological analyses have identified it as the source of Tahiti's *Partula *species [35], although there may have been some back-migration from Tahiti to southern Moorea [26]. Our goal therefore was to build a combined Moorean/Tahitian phylogeny that incorporated extant wild populations, extant captive populations and historical museum samples, the latter collected by J.B. Burch in 1970, prior to the introduction of *Euglandina rosea*.

A recent phylogenetic study of Tahitian wild, captive and historical museum tree snails found that at least some members of all five primary historical Tahitian *Partula *mt clades remain extant, primarily in montane refuge populations [36]. This result was somewhat unexpected, given that three of Tahiti's eight *Partula *species are extinct [13]. However, Society Island partulid taxonomic designations are predominantly conchological [20,21], and they are often poorly-corroborated by molecular markers, although this is further complicated by incongruence among genetic marker sets [25-28,36,37]. In this present study, we were particularly interested in testing for cryptic genealogical links among surviving Windward Island partulid populations on Moorea and on Tahiti. Our results show that although much of Moorea's tree snail mitochondrial diversity (including one of the primary tip clades) has been lost, a surprisingly representative genealogical sub-sample collectively persists in captivity, in the wild on Moorea and, in the form of five phylogenetically distinct sister lineages, on Tahiti.

## Results and discussion

### Windward Island Overview

Combining our novel data (82 distinct haplotypes from 161 snails) with preexisting mt COI datasets [30,36,37] produced a total Windward Island genotyped partulid sample size of 457 snails. Although this malacofauna has experienced extreme extinction pressure in recent decades, access to museum and captive specimens enabled us to incorporate genotypes from six extinct species (three Moorean) as well as from the five extirpated species (four Moorean) that persist in captivity [13]. Consequently, we have almost complete nominal species taxonomic representation: 16 out of the 17 Windward Island taxa listed in a recent study [13] in addition to the endemic Tahitian *Samoana burchi*. The missing species, *Partula cytherea*, has not been seen since its 1920's discovery on a remote Tahitian interior mountain slope [38] and it is now presumed extinct [13].

**Figure 1:**
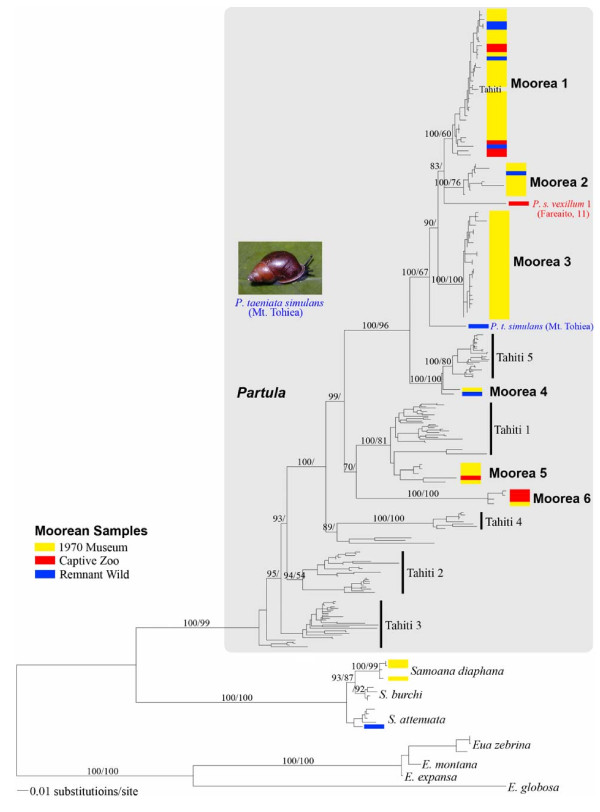
**Maximum likelihood tree showing an overview of the inferred phylogenetic relationships of Windward Island partulid mt COI genotypes**. The partulid genus *Eua*, restricted to central Pacific islands in Samoa, American Samoa and Tonga, served as the outgroup [42] and the grey background applied to the *Partula *clade visually distinguishes the two ingroup genera. *Partula *tip clades were taxonomically polyphyletic and the six Moorean tip clades are numbered in bold, as were the five Tahitian clades previously characterized [36]. Terminal Moorean haplotypes are color-coded according to their sample source: Burch 1970 Museum specimens; captive snails; extant wild populations. Two divergent Moorean mt haplotypes were recovered from single snails and are labeled individually: a captive *P. suturalis vexillium *and an extant wild Mt. Tohiea *P. taeniata simulans *(identified by J. Murray). A photograph of the latter specimen, taken in the field by J.Y. Meyer, is inserted. Support levels are given above major internal branches; Bayesian posterior probabilities (> 70) on the left and maximum parsimony bootstrap support values (> 50) on the right.

Figure 1 gives an overview of the Windward Island partulid mt COI phylogeny recovered. The most trenchant topological feature was the well-supported reciprocal monophyly of *Samoana *and *Partula *taxa, which corroborated numerous lines of anatomical [33,39], allozymic [35,40] and nuclear rDNA [41,42] evidence of their distinctiveness. In the *Partula *clade, the basal Windward Island lineages were exclusively Tahitian, and the Moorean haplotypes formed six tip clades, three of which (Moorea 1–3) contained the large majority of the island's haplotypic diversity and occupied the most derived portion of Windward Island tree space (Figure 1). The remaining three Moorean tip clades (Moorea 4–6) were interspersed with Tahitian lineages, and two of them formed robust exclusive sister relationships with Tahitian tip clades: Moorea 4 and Tahiti 5; Moorea 5 and Tahiti 1. The most robust internal stem branch supported approximately half of the *Partula *clade treespace (Moorea 1–4; Tahiti 5) and, with the exception of *P. nodosa *(Tahitian clade 3; [36]), its taxonomic composition was consistent with Murray *et al's *[25] most common Windward Island *Partula *mt RFLP genotype (A). The absence of major clades exclusive to captive and wild samples (Figure 1) implied that the reference museum collection may be broadly representative of pre-collapse 1970 Moorean *Partula *mt diversity. Nevertheless, two divergent extant haplotypes (one from a wild *P. taeniata *on the summit of Mt. Tohiea; the other from a captive *P. s. vexillium *sampled in Fareaito) lack 1970 phylogenetic matches (Figure 1). Detailed discussion of each Moorean tip clade follows below.

### Moorean Clade 1

**Figure 2:**
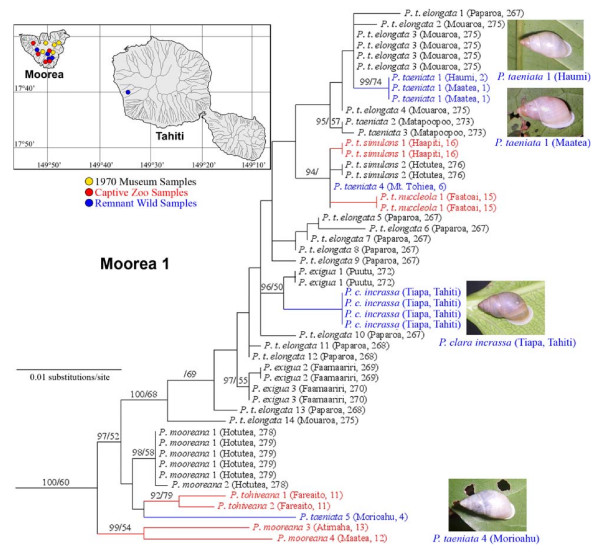
**A detailed view of Moorean Clade 1 (Figure 1) mt COI genotypes**. The taxonomic identity of each haplotype is given together with its geographic origin in Moorea, or Tahiti (valley name and sampling location code). The taxonomic labels are color-coded according to source: Burch 1970 museum specimens; captive snails; extant wild populations. The insert map shows the geographic origins of museum, captive and extant Clade 1 mt genotypes obtained from 14 locations throughout Moorea and from a single location (Tiapa Valley) in Tahiti. Insert photographs, taken in the field by T. Coote, are shown for three surviving populations of *P. taeniata *on Moorea and the single population of *P. clara incrassa *on Tahiti. Support levels are given above branches; Bayesian posterior probabilities (> 70) on the left and maximum parsimony bootstrap support values (> 50) on the right.

Figure 2 gives a fine-scale view of Moorean Clade 1, showing the taxonomic identity and the geographic origin of each constituent snail genotyped for mt COI. Focusing first on Moorean haplotypes, this clade was taxonomically polyphyletic, but in a topologically structured manner. Derived Moorean lineages, comprising approximately 80% of this clade's topology, were exclusively composed of *Partula taeniata *(widely distributed across the island) and *P. exigua *(Figure 2). These two species readily hybridize under laboratory conditions [22] and collectively form the *P. taeniata *species complex [23]. Clade 1 basal lineages were sourced from the south central part of the island and most were obtained from *P. mooreana *and *P. tohiveana*, both members of the *P. suturalis *species complex [23]. This basal segment of Clade 1 treespace was broadly congruent with the *P. taeniata/P. mooreana/P. tohiveana *mt Cytochrome B clade previously recovered from many of the same southern localities [34]. Although *P. exigua *is extinct, *P. taeniata *Moorean Clade 1 lineages persist both in captivity and in three central/south island wild populations: Haumi and Maatea valleys, as well as on upper slopes of Mt. Tohiea (Figure 2). Basal Clade 1 lineages of *P. mooreana *and *P. tohiveana *survive only in captivity but a remnant wild *P. taeniata *population persists in Morioahu valley (Figure 2).

Moorean Clade 1 lineages initially appeared to be restricted to that island because no constituent haplotypes had been encountered in Lee *et al*'s [36,37] extensive genotyping of Tahitian wild, captive and historical museum tree snails. However, four Tahitan snails from a recently discovered extant population of *Partula clara *in Tiapa Valley (unsampled by Burch in 1970) all shared a haplotype that surprisingly nested firmly within Moorean Clade 1, sister to a Moorean Puutu Valley population of *P. exigua *(Figure 2). Crampton recognized the Tiapa (Aoua, in his terminology) Valley population as a distinct subspecies, *P. clara incrassa*, stating that it was extraordinarily distinct from nominal conspecifics in adjacent valleys in that it had *longer thinner shells, peculiar color morphs including unique banded mutants, thinner shells and lips and some specimens with a trace of a pillar tooth *[20]. Our extant wild Tiapa Valley snails are conchologically indistinguishable from Crampton's voucher specimens of *P. c. incrassa *(see Additional File 1) and the haplotype they carry is phylogenetically distinct from all other Tahitian partulids (Figure 1), including members of the primary *P. clara/hyalina *clade in adjacent valleys [37]. Snails bearing Moorean Clade 1 mitochondria therefore appear to have established a discrete founder population in this Tahitian valley, where they now represent the only surviving partulids (T. Coote pers. observ.), and this clade remains tenuously extant on both Moorea (*P. taeniata*) and Tahiti (*P. c. incrassa*), as well as in captivity.

### Moorean Clade 2

**Figure 3:**
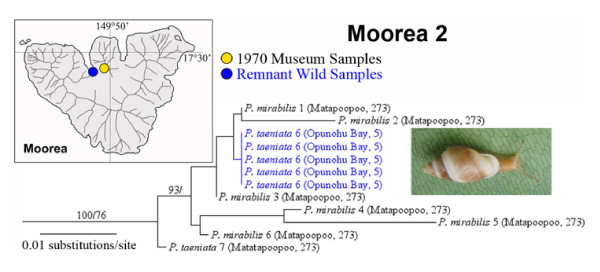
**A magnified view of Moorean Clade 2 (Figure 1) mt COI genotypes**. The taxonomic identity of each haplotype is given together with its geographic origin in either Matapoopoo Valley (Burch 1970 museum samples) or the Opunohu Bay mangrove fern locality (extant population). The insert photograph, taken in the field by C. Hickman, shows one of the 5 surviving wild *P. taeniata *that were biopsied and sequenced. Support levels are given above branches; Bayesian posterior probabilities (> 70) on the left and maximum parsimony bootstrap support values (> 50) on the right.

Prior to the introduction of *Euglandina rosea*, three Moorean species (*Partula taeniata*, *P. suturalis *and *P. mirabilis*) had two areas of sympatry, the smaller occurring on the Mt. Rotui Peninsula [22,23]. We obtained a well-supported and phylogenetically distinctive mt clade (Moorea Clade 2) from a sub-sample of 1970 Mt. Rotui Peninsula (Matapoopoo Valley) *P. mirabilis *and *P. taeniata *specimens (Figure 3). This polyphyletic result was unsurprising because *P. mirabilis *can hybridize with both *P. taeniata *and *P. suturalis *species complexes [22]. Moorean Clade 2 appears to have been restricted to the Mt. Rotui Peninsula (Figure 3) and was not represented in the captive populations, none of which were sourced there. However, this mt lineage remains precariously extant; five biopsies from a nearby surviving population of *P. taeniata*, discovered by C. Hickman in 2002, all produced a Clade 2 haplotype (Figure 3). These survivors persist in an unusual mangrove fern micro-habitat fringing Opunohu Bay, exhibit a variety of shell-color morphs, and may be protected from *Euglandina rosea *incursions by a brackish water moat (C. Hickman unpubl. observ.).

### Moorean Clade 3

**Figure 4:**
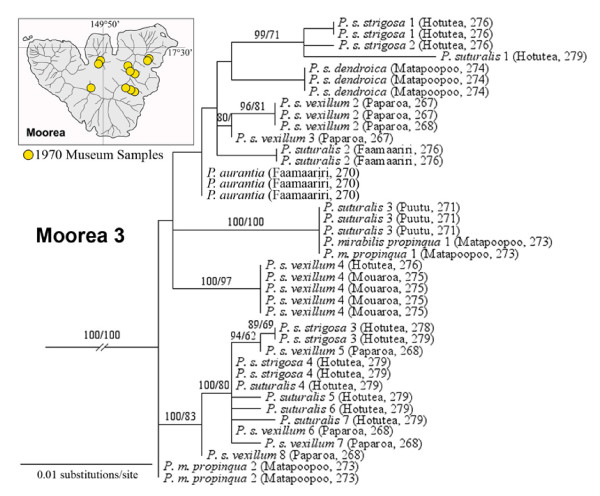
**A detailed view of Moorean Clade 3 (Figure 1) mt COI genotypes**. This tip clade was exclusively composed of museum specimens sampled by J. B. Burch in 1970 from 11 sampling stations (see insert map). The taxonomic identity of each haplotype is given together with its geographic origin. Support levels are given above branches; Bayesian posterior probabilities (> 70) on the left and maximum parsimony bootstrap support values (> 50) on the right.

This major endemic Moorean clade was recovered from 11 out of 13 Burch 1970 sampling stations and was present in high frequencies in central and eastern parts of the island (Figure 4). Moorean Clade 3 was almost exclusively (36/40 snails) composed of *P. suturalis *(including nominal subspecies) and *P. aurantia*, both members of the *P. suturalis *species complex [23,32] that readily hybridized in the laboratory [22]. The other Clade 3 taxon consisted of four Matapoopoo Valley specimens of *Partula mirabilis propinqua *[22], two of which shared a haplotype with Puutu Valley *P. suturalis *specimens (Figure 4). A notable feature of this clade was the conspicuous absence of *P. mooreana *and *P. tohiveana*, the remaining two members of the *P. suturalis *species complex [23]. They placed instead within Moorean Clade 1, together with members of the *P. taeniata *species complex (Figure 2; [34]). It may be pertinent that *P. mooreana *and *P. tohiveana *showed little or no ability to hybridize with *P. suturalis *under laboratory conditions [22]. Those reproductive incompatibilities, coupled with their observed mt phylogenetic distinctiveness (Figures 2, 4; [34]), undermine the case for inclusion of *P. mooreana *and *P. tohiveana *in the *P. suturalis *species complex.

Both *Partula aurantia *and *P. m. propinqua *are now presumed extinct. Two *P. suturalis *subspecies survive in captivity. They were originally sourced from one central valley (*P. s. vexillum*: Fareaito; see topological placement in Figure 1) and two southern valleys (*P. s. vexillum*: Vaianai; *P. s. strigosa: *Maatea), but none of the genotyped captive specimens bore Moorean Clade 3 mt lineages (Figure 4). It would appear that Moorean Clade 3, a major endemic component of the island's historical partulid mt tree space, may well be extinct.

### Moorean Clade 4

**Figure 5:**
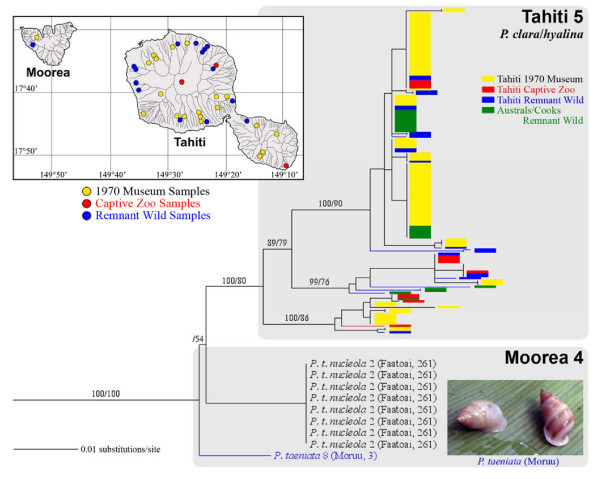
**Topological, taxonomic and distributional details of Moorean Clade 4 (Figure 1), and its sister clade, Tahiti 5 **[36]. Moorean Clade 4 haplotypes were recovered from *P. taeniata *individuals in two northwestern valleys (see insert map) and an insert photograph, taken in the field by T. Coote, is shown of surviving Moruu Valley specimens. Snails bearing Tahiti Clade 5 haplotypes remain extant on Tahiti (composed of *P. hyalina *and *P. clara *[36,37]) and on the Cook and Austral archipelagoes (*P. hyalina *only [37]). Support levels are given above branches; Bayesian posterior probabilities (> 70) on the left and maximum parsimony bootstrap support values (> 50) on the right.

Eight individuals of *Partula taeniata nucleola *sampled from a northwestern Moorean valley (Faatoai) by J. B. Burch in 1970 all carried a haplotype (Moorean Clade 4; Figure 5) that was phylogenetically divergent from all other genotyped Moorean museum and captive partulids, including captive *P. t. nucleola *sourced from that same valley (Moorean Clade 1; Figures 1, 2). Goodacre also recovered two divergent *P. taeniata *mt haplogroups from independent historical Faatoai Valley samples [27]. One haplogroup was relatively rare in Faatoai, but widespread throughout the island, as is Moorean Clade 1 (Figure 2); the other was predominant in western valleys, including Faatoai and Moruu [27]. A newly discovered remnant Moruu Valley population of *P. taeniata *provided the first Moorean phylogenetic match to the Burch 1970 *P. t. nucleola *lineage (Moorean Clade 4; Figure 5). We therefore consider it likely that our Moorean Clade 4 and Goodacre's *P. taeniata *western haplogroup [27] both represent the same lineage and that, though absent from captive populations, it still remains extant in Moruu Valley.

Viewed from a single-island perspective, Moorean Clade 4 seems relatively unimportant: it lacks on-island sister lineages, represents a minor component of the island's historical partulid mt tree space, had a restricted original distribution and is not represented in captive populations (Figures 1, 5; [27]). However, taking a multi-island perspective revealed these Moorean *Partula taeniata *mt lineages to be part of a larger mt clade with a substantial Windward Island, and regional archipelagic distribution. Moorean Clade 4 has a robust and exclusive sister relationship with a major Tahitian mt lineage comprising two nominal species, *Partula hyalina *and *P. clara *(Tahitian Clade 5; Figures 1, 5; with the exception of the Tiapa Valley population of *P. c. incrassa*, Figure 2). Tahitian *P. hyalina *and *P. clara *snails have also proven to be differentially resistant to *Euglandina rosea *predation. Although these two nominal taxa collectively represented only *circa *5% of historical Tahitian valley *Partula *spp. populations [20], they now form 100% of the surviving populations in numerous Tahitian valleys [14], the latest estimate being 33 valleys (T. Coote, unpubl. observ.). Multiple 1970-era Tahitian haplotypes were recovered from genotyped extant wild and captive Tahitian *P. hyalina *and *P. clara *snails and also from extant *P. hyalina *anthropogenic founder populations in the Cook and Austral Islands, two neighboring hot spot archipelagoes (Figure 5; [36,37]). Regional wild snail populations bearing members of this phylogenetically-distinctive mt lineage (Moorea 4 + Tahiti 5) therefore survive tenuously in the presence of *Euglandina rosea *on Moorea and on Tahiti and thrive in its absence on the Cook and Austral archipelagoes (Figure 5; [37]).

### Moorean Clade 5

**Figure 6:**
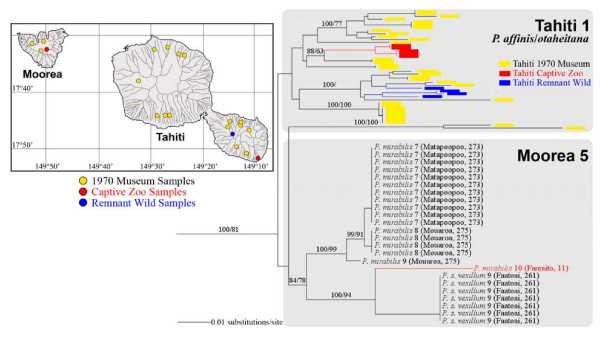
**A detailed view of Moorean mt Clade 5 (Figure 1) and its paraphyletic sister mt clade, Tahiti 1 **[36]. The taxonomic identity of each Moorea Clade 5 haplotype is given together with its geographic origin and sample source: Burch 1970 Museum *P. mirabilis *and *P. suturalis vexillium *or captive *P. mirabilis*. Tahitian Clade 1 lineages were composed of *P. otaheitana *and *P. affinis *[36]. Support levels are given above branches; Bayesian posterior probabilities (> 70) on the left and maximum parsimony bootstrap support values (> 50) on the right.

A second phylogenetically-distinct Moorean mt lineage (Moorean Clade 5) with a robust Tahitian sister clade was recovered from museum samples of *Partula suturalis vexillium *and from museum and captive samples of *P. mirabilis*, all sourced from northwest/central valleys (Figure 6). The *P. s. vexillium *snails from the northwestern valley Faatoai placed firmly in Moorean Clade 5 (Figure 6), unlike their Moorean Clade 3 conspecifics sampled across the northcentral and northeast of the island (Figure 4). Moorean Clade 5 museum samples also contained *P. mirabilis *from both of its geographically-disjunct [22] populations: Mt. Rotui (Matapoopoo Valley) and central Moorea (Mouaroa Valley). Murray and Clarke found that both *P. mirabilis *populations hybridized readily [22], and this is consistent with our finding of a very close mt phylogenetic association among this taxon's Matapoopoo and Mouaroa clade 5 snails (Figure 6). Nevertheless, it should be kept in mind that the Matapoopoo *P. mirabilis *1970 sample was highly heterogeneous in its mt lineage composition. It also contained snails with Moorean Clade 2 haplotypes (Figure 3; shared with *P. taeniata*) as well as *P. m. propinqua *individuals bearing Moorean clade 3 haplotypes (Figure 4; shared with *P. suturalis *and *P. aurantia*). These results corroborate Murray and Clarke's view [22] of *P. mirabilis *as a species that could hybridize with both *P. taeniata *and *P. suturalis *species complexes.

Moorean Clade 5 is now apparently extirpated in the wild, but a constituent haplotype survives in a captive *P. mirabilis *population, originally sourced from Fareaito Valley (Figure 6). Taking a multi-island perspective, Moorean Clade 5 is part of a larger and phylogenetically-distinctive Windward Island mt lineage that incorporates an exclusive Tahitian sister clade: Tahiti 1 (Figures 1, 6; [36]) and that collectively has a taxonomic composition consistent with Murray *et al's *[25] mt RFLP genotype "P". Tahiti Clade 1 contained two nominal species, *P. affinis *and *P. otaheitana*, had an island-wide distribution, and two known populations persist: *P. affinis *in captivity and a remnant montane population of *P. otaheitana *on Mt. Atara (Figure 6; [36]). Unlike the two other nested Moorean/Tahitian *Partula *spp. clades (Figures 2, 5), snails bearing this multi-island mt lineage appear incapable of surviving in Windward Island valleys in the presence of *Euglandina rosea*.

### Moorean Clade 6

**Figure 7:**
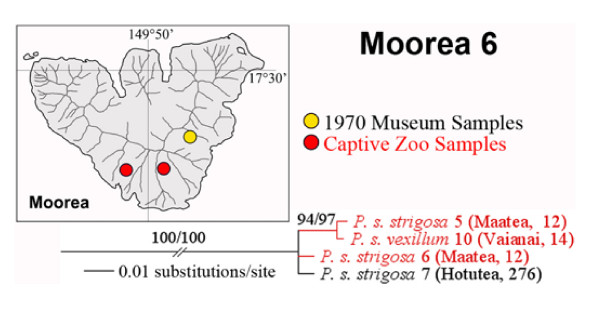
**Topological, taxonomic and distributional details of Moorean Clade 6 (Figure 1) haplotypes**. They were obtained from Burch 1970 museum and captive samples of *Partula suturalis strigosa *and captive *P. s. vexillium*. Support levels are given above branches; Bayesian posterior probabilities (> 70) on the left and maximum parsimony bootstrap support values (> 50) on the right.

Murray *et al*. [25] found that southern Moorean populations of *Partula suturalis *had a highly distinctive mtDNA RFLP genotype that differed markedly from conspecific northern populations. Our limited sampling of southern *P. suturalis *confirmed this result. We recovered a divergent tip clade (Moorea Clade 6; Fig 7) from captive lines of two subspecies sourced from Maatea and Vaianai Valleys, together with a single *P. s. strigosa *snail sampled in 1970 from Hotutea Valley (Figure 7). The other four 1970 Hotutea *P. s. strigosa *individuals genotyped from that same sampling station (276) placed in Moorean Clade 3 (Figure 4). Moorean Clade 6 appears to have been extirpated in the wild but persists in captivity.

### Samoana

**Figure 8:**
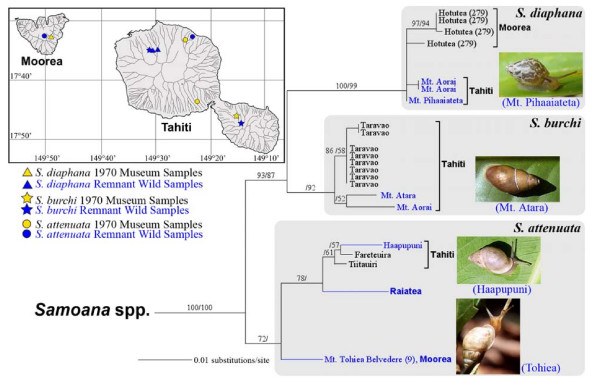
**A magnified view of the *Samoana *mt COI clade (Figure 1)**. It shows the topological placement and distribution of Burch 1970 museum specimens together with those of extant wild samples. These include *S. diaphana *(Moorea and Tahiti, photograph of extant Tahitian snail by B. Holland); *S. burchi*, (Tahiti only, photograph of extant snail by T. Coote) and *S. attenuata *[Moorea and Tahiti, photographs of extant wild snails by C. Hickman (Moorea) and T. Coote (Tahiti)]. A mt genotype obtained from a surviving Raiatean population [30] is also included. Note that *S. attenuata *is known to persist in additional Tahitian valleys [14]. Support levels are given above branches; Bayesian posterior probabilities (> 70) on the left and maximum parsimony bootstrap support values (> 50) on the right.

Three allozymically-corroborated Windward Island *Samoana *morphospecies have been recorded [33,43]: *S. attenuata *(Moorea and Tahiti); *S. diaphana *(Moorea and Tahiti) and *S. burchi *(Tahiti only). No captive populations exist but lyophilized 1970 museum tissue was available for all three species, although from single island populations only of *S. attenuata *(Tahiti) and *S. diaphana *(Moorea). Genotyped museum samples were phylogenetically analyzed together with sequences obtained from tissue biopsies of surviving *S. attenuata *populations on Tahiti and Moorea [14] and on Raiatea [30], together with recently discovered extant Tahitian montane populations of *S. diaphana *and *S. burchi*. Our phylogenetic results confirm that *S. attenuata *survives on Moorea and that all three taxa persist in the wild on Tahiti (Figure 8; but see also Additional File 2 for possible persistence of *S. diaphana *on Moorea). Individual haplotypes were not shared among the multi-island samples of *S. attenuata *and *S. diaphana*, and the former species did not form a Windward Island clade: Tahitian haplotypes instead clustered with the Raiatean sample.

Topological details of the *Samoana *clade also corroborated earlier allozyme studies that revealed strikingly different phylogenetic profiles among co-occurring *Samoana *and *Partula *taxa [43]. These distinctions included the much lower collective genetic diversity levels of *Samoana *species (compare relative generic treespaces in Figure 1) and their more pronounced mt phylogenetic cohesiveness (all three *Samoana *species were reciprocally monophyletic, including the multi-island taxa; Figure 8). *Samoana *and *Partula *lineages therefore appear to have experienced quite distinct patterns of cladogenesis in the Windward Islands and, post *Euglandina rosea *introduction, they also have experienced differential patterns of extirpation and survival. This is a particularly interesting result, given that these three taxa were originally much scarcer than co-occurring *Partula *species in the Society Islands [33].

## Summary discussion

One of the primary challenges faced in constructing a meaningful phylogeny of a largely extirpated fauna concerns the issue of comprehensiveness; how confident are we that our historical reference samples contain the primary Moorean endemic lineages? Although our taxonomic sample of Windward Island Partulidae was almost complete, this in itself was insufficient because of the poor correlation of nominal taxonomy with molecular markers [26]. Nevertheless, our novel results were in good agreement with previous independent mt characterizations of these taxa [25,27,28,34], both within Moorea (*e.g*., geographic distributions and taxonomic composition of the primary clades) and among the two islands, apart from a small number of relatively minor issues such as the presence of the Tahitian species *Partula nodosa *within the primary Windward Island mt RFLP lineage [25]. We are therefore confident that our historical reference Moorean dataset probably contains the primary Moorean lineages and that it can be used to assess what fraction of the island's endemic mt treespace has survived in captivity and/or in the wild.

Our Windward Island dataset yields a novel, and somewhat complex, multi-island genealogical perspective on Moorean partulid survival. Only one of eight historical Moorean partulid tip clades (six *Partula *spp.; two *Samoana *spp.) is extinct. Unfortunately, the extinct lineage (Moorea Clade 3) encompassed the bulk of the *P. suturalis *species complex, and lacked Tahitian sister lineages (Figures 1, 4). The seven extant clades exhibited a heterogeneous spectrum of persistence: on Moorea, in captivity and also on Tahiti (Moorean Clade 1); on Moorea, with sister lineages on Tahiti, on other archipelagoes, and in captivity (Moorea Clade 4); on Moorea, with sister lineages on Tahiti (*S. attenuata*); on Moorea (Moorea Clade 2); in captivity, with sister lineages on Tahiti and also in captivity (Moorea Clade 5); sister lineages on Tahiti (*S. diaphana*); in captivity (Moorea Clade 6).

Our assessment of Moorean partulid survival comes with an obvious caveat concerning its broader utility; do these results have relevance to understanding historical Moorean partulid whole organism genealogies, and their present day conservation status? Fortunately, this did not appear to be an issue for Windward Island *Samoana *species where there was excellent taxonomic/mt lineage congruence (Figure 8). However, Society Island partulid taxonomy may be poorly corroborated by molecular markers and, in addition, different nuclear (allozymes and nRNA) and organellar genetic marker sets may also be incongruent [25-28,36,37]. Although we cannot address this issue directly using allozymes or high-resolution nuclear markers, two lines of indirect data indicate that our museum, captive and remnant wild mt genealogies do have broader biological and conservation significance. First, the taxonomic composition of our clades is in good agreement with the results of Moorean breeding experiments [22], *e.g*., reproductive compatibility of *P. taeniata *and *P. exigua*; compatibility of *P. suturalis *and *P. aurantia*; incompatibility of *P. mooreana *and *P. tohiveana *with *P. suturalis*, compatibility of *P. mirabilis *with both *P. taeniata *and *P. suturalis*. Second, the ability of *Partula *spp. populations to persist on Moorea and in Tahitian valleys in the long-term presence of *Euglandina rosea *appears to be correlated with both taxonomy and mt phylogeography.

Gerlach [44] proposed that impaired predator performance at altitude would allow the persistence of Society Island partulids in montane refuges and we see evidence of this in Tahiti where substantial montane populations preserve multiple mt clades that have been extirpated at lower altitudes

**Figure 9:**
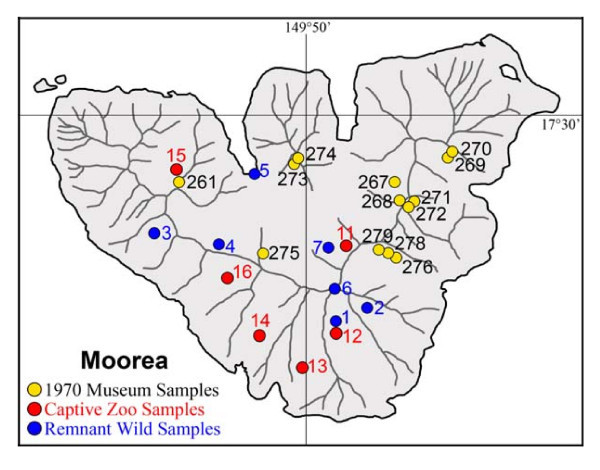
**Map of the island of Moorea showing partulid sampling locations**. Interior lines represent major ridges. The labeling code for J. B. Burch's 1970 sampling stations was as follows: **261**: Faatoai Valley, northern slope of Mt. Tautuapae (350 m altitude); **267**: Paparoa V. (100 m); **268**: Paparoa V. (300 m); **269**: Faamaariri V. (150 m); **270**: Faamaariri V. (300 m); **271**: Puutu V. (250 m); **272**: Puutu V. (400 m); **273**: Matapoopoo V. (150–200 m); **274**: Matapoopoo V. (400 m); **275**: Mouaroa V. (200–300 m); **276**: Hotutea V. northeastern ridge (400 m); **278**: Hotutea V. northeastern ridge (480 m); **279**: Hotutea V. northeastern ridge (620 m). Extant wild populations were detected and biopsied in the following locations: **1**: Maatea Valley (180 m; June 2007); **2**: Haumi V. (June 2007); **3**: Moruu V. (June 2005); **4**: Morioahu V. (September 2007); **5**: Opunohu Bay mangrove (November 2006); **6**: Mt. Tohiea (1150 m; January 2002 and September 2006); **7**: *Partula *Reserve, Mt. Tohiea Belvedere (October 2006). Captive zoo populations were sourced from the following valleys (exact locations within valleys are uncertain): **11**: Fareaito V.; **12**: Maatea V.; **13**: Atimaha V.; **14**: Vaianai V.; **15**: Faatoai V.; **16**: Haapiti V.

[36]. Moorea has a much smaller montane (> 1000 m) habitat than Tahiti and there is no part of the island where a representative sampling of the original *in-situ *diversity (taxonomic and/or mt lineage) has survived. This can be readily visualized by comparing the distribution pattern of surviving *Partula taeniata *populations (Figure 9; locations 1–6) with the pre-*Euglandina rosea *introduction distribution maps of the island's species of *Partula *[23]. Although the two widespread Moorean species, *Partula taeniata *and *P. suturalis*, originally had almost identical over-lapping distributions on Moorea [23], all six known surviving populations of Moorean *Partula *are exclusively composed of *P. taeniata*. Differential survival is also evident among mutually exclusive mt clades: Moorean Clades 1, 2 and 4 for *P. taeniata *(all of which persist in the wild); Moorean Clades 3, 5 and 6 for *P. suturalis *(all of which are apparently extirpated in the wild). There is no apparent correlation of survival with altitude on Moorea: extant *P. taeniata *are found at sea-level (Opunohu Bay), at lower altitudes (Maatea Valley, 180 m), and also just below (1150 m) the summit (1207 m) of Mt. Tohiea, the highest point on the island (Figure 1).

Predation models predict extirpation of *Partula *spp. populations within 3 years of initial *Euglandina rosea *contact [45]. Snails bearing four Moorean mt lineages (Clades 2, 3, 5 (+ its sister clade Tahiti 1) and 6) meet this prediction in that they have not persisted in the presence of the predator, surviving in the wild only in insulated micro-habitats such as the Opunohu Bay mangrove fern enclave (Moorea Clade 2; Figure 3) or in a high altitude montane refuge (Tahiti Clade 1; Figure 6; [36]). In contrast, snail populations bearing one of two multi-island mt lineages (Moorea Clade 1; Moorea 4 & Tahiti 5) have successfully survived three decades of direct exposure to the predator on Moorea and in many Tahitian valleys.

It is unclear at present what biological attributes, genetic and/or ecological, underlie this differential persistence: Crampton [20,21] documented relatively higher fecundities in *Partula hyalina *and *P. clara *(Tahiti Clade 5), but not in *P. taeniata *(Moorean Clades 1, 4). Detailed field studies of the surviving populations are urgently required and these may yield clues toward developing a viable long-term conservation plan for Society Island partulid tree snails. Goodacre's mt population study of *P. taeniata *historical populations documented the presence of a pronounced cline involving a western haplogroup (probably our Moorean Clade 4) and an island-wide haplogroup (probably our Moorean Clade 1) in northwestern valleys that was not corroborated by variation in 6 polymorphic allozyme loci [27]. It is therefore possible that surviving *P. taeniata *populations bearing these two mt clades may share common sets of hypothetical nuclear genome-encoded "resistance" traits, but this remains to be determined. Irrespective of the underlying mechanisms, the differential resilience exhibited by *P. taeniata*, *P. hyalina *and *P. clara *identifies them as the most promising captive candidate lineages for future Windward Island reestablishment attempts.

## Conclusion

Conservation efforts directed toward Moorean and Tahitian partulids have typically operated within a single island frame of reference [14,18,29]. However, there is an increasing appreciation among conservation biologists for the importance of evolutionary and ecological processes in effective conservation planning [46] and the multi-island genealogical relationships of Moorean partulid taxa (Figure 1), specifically the presence of five phylogenetically-distinct sister lineages on Tahiti, provide a broader Windward Island evolutionary perspective on Moorean tree snail survival. Our results imply that, for many endemic partulid lineages, it may be apt to consider Moorea and Tahiti in a more integrated fashion, where proposed conservation initiatives on one of the islands are evaluated within a collective Windward Island genealogical context. This may be especially pertinent in the case of the genealogically-linked surviving lineages of *P. taeniata*, *P. hyalina *and *P. clara*, but it is also potentially relevant for conservation measures involving Thaitian montane refuges where snails with robust phylogenetic ties to extirpated Moorean lineages still persist (Figure 6). Multi-island genealogical perspectives will probably prove to be of proactive conservation value not only for Windward Island partulids, but also for the critically endangered terrestrial biotas of many other Pacific hot spot archipelagoes [47,48].

## Methods

### Sampling

**Figure 10:**
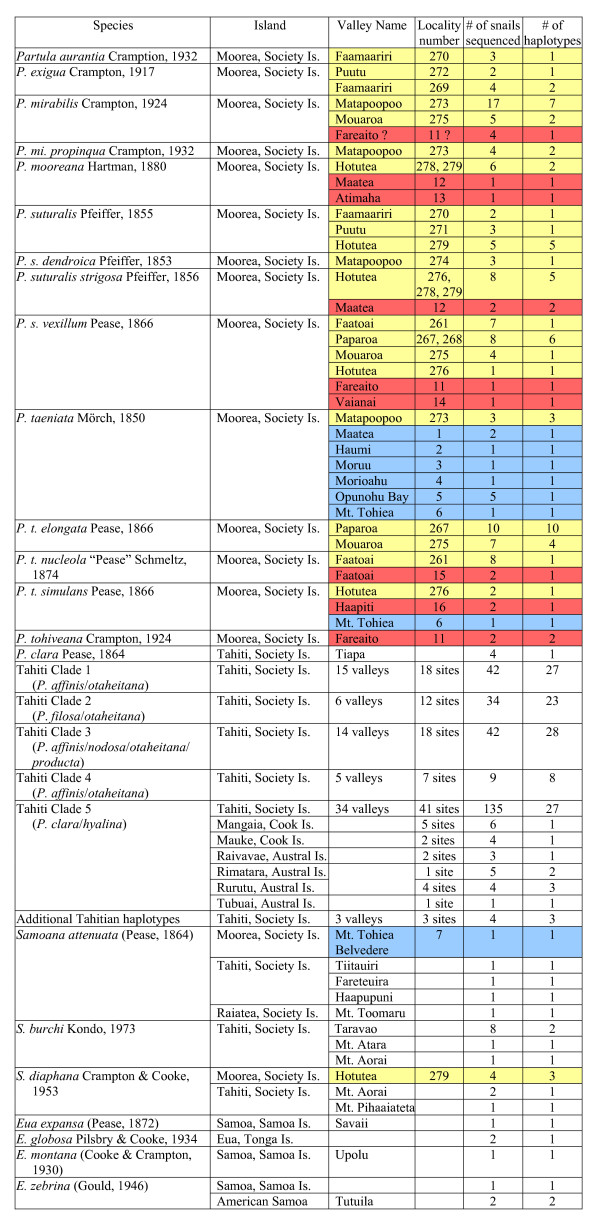
**Taxonomic designations and sampling locations for Moorean partulids**. Moorean samples are shaded in yellow (Burch 1970 museum), red (captive zoo) and blue (remnant wild). Summary data are also provided for non-Moorean partulids [30,36,37] employed in the study. See Additional File 3 for detailed information, including museum voucher and GenBank numbers, on all the snails (Moorean and non-Moorean) incorporated into the summary phylogenetic tree (Figure 1).

See Figure 10 for an overview of the sampling locations and the taxonomic identity of partulid snails genotyped in this study. Voucher information is available in Additional File 3 together with sampling details for previously genotyped partulid snails [30,36,37] incorporated into the phylogenetic analyses.

J. B. Burch and associates sampled Moorean partulids in October 1970 at 13 stations (Figure 9). Specimens of all nominal Moorean partulid species except for *Partula tohiveana *and *Samoana attenuata *were airmailed alive to the University of Michigan Museum of Zoology (UMMZ) where foot tissues were lyophilized and shells retained as vouchers. We selected 125 lyophilized Moorean individuals for genotyping based on Crampton's species and subspecies-level taxonomy (Figure 10; Additional File 3). From 1980 to 1985, emergency field sampling of six populations (Figure 9) led to the establishment of a number of captive Moorean tree snail lines [15,17]. Sixteen captive snails representing five Moorean *Partula *species [*P. mirabilis*, *P. mooreana*, *P. tohiveana*, *P. taeniata *(subspecies *P. t. nucleoli*, and *P. t. simulans*) and *P. suturalis *(subspecies *P. s. vexillum *and *P. s. strigosa*)] were supplied by the International Partulid Conservation Programme for genotyping. From 2002–2006, a small number of biopsied tissue samples were obtained from extant Moorean populations, preserved in 95% ethanol and forwarded for genotyping (Figure 10). These included a specimen of *Samoana attenuata *from immediately below the Afareaito *Partula *Reserve in the upper Oponuhu Valley and specimens of newly discovered *Partula taeniata *populations from a variety of locations: Maatea, Haumi and Moruu valleys; a mangrove fern habitat in Opunohu Bay; and two temporally distinct (2002, 2006) samples from just below (1150 m) the summit (1207 m) of Mt. Tohiea, the highest point on the island (Figure 9, Additional File 3). Biopsies from newly discovered extant Tahitian populations of *P. clara *(Tiapa Valley), *S. attenuata *(Haapupni Valley) and putative *S. burchi *(Mt. Atara, Mt. Aorai) and *S. diaphana *(Mt. Aorai, Mt. Pihaaiateta) that were not included in previous studies [36,37] were also genotyped.

### Molecular Data

Total genomic DNA was isolated using a DNeasy Tissue Kit (Qiagen) according to the manufacturer's instructions. A 655 nucleotide (nt) mt COI target fragment was amplified with GoTaq DNA Polymerase (Promega, Madison, WI) using the ''universal'' primer pair LCO1490/HCO2198 [49] and a negative control (no template) was included in each amplification run. After 2 min denatuation at 95°C, an initial annealing temperature of 65°C was decreased by 2°C/cycle (30 sec denaturing at 95°C, 40 sec annealing and 1 min extension at 72°C) until the final annealing temperature (45°C) was reached and subsequently maintained for an additional 30 cycles. Double-stranded products were isolated on 1% agarose gels, excised over UV light, and extracted using a QIAquick gel extraction kit (Qiagen, Valencia, CA). Both strands of the amplified fragments were directly cycle-sequenced, using the PCR primers, by the University of Michigan's Sequencing Core Facility. All DNA sequences obtained have been deposited in GenBank (EU832996–EU833099).

### Phylogenetic analyses

The resulting chromatograms were edited by comparing both strands using Sequence Navigator 1.0.1 (Applied Biosystems, Foster City, CA). COI sequences were aligned easily due to an absence of indels. Maximum likelihood (ML) analyses were performed using PAUP*4.0b10 [50] under the TVM+I+G model of sequence evolution, the best-fit model selected by Akaike Information Criterion implemented in Modeltest 3.7 [51]. Likelihood parameters [base frequencies (A = 0.3300, C = 0.1064, G = 0.1354, T = 0.4282); rate matrix (1.1933, 27.6197, 1.8944, 0.4618, 27.6197, 1); shape of gamma distribution = 1.2494; proportion of invariable sites = 0.5742] found in Modeltest were used and heuristic searches were employed by using a neighbor-joining starting tree and nearest neighbor interchange (NNI) branch swapping. The partulid genus *Eua*, restricted to central Pacific islands in Samoa, American Samoa and Tonga, was used as the outgroup [42]. Initial searches found two ML trees with a log-likelihood (ln L) value of -9968.9197. Both trees were further used as starting trees for another round of heuristic search and a single ML tree (ln L = -9967.6214) was recovered.

Bayesian posterior probabilities and parsimony bootstrap were employed to measure nodal support. Parsimony bootstrapping [52] was done with the "fast" stepwise-addition option for heuristic searches (1000 replicates) using PAUP*. Bayesian analyses were performed using MrBayes 3.1.2 [53] set for the GTR+Γ+I model. Model parameters were treated as unknown and were estimated for each analysis. Four chains were run simultaneously for 1,000,000 generations and trees were sampled every 100 cycles. Posterior probability values were estimated by generating a 50% majority rule consensus tree after the burn-in period of 2,000 using PAUP*.

## Authors' contributions

DÓF and TL conceived and designed the study; TL generated the sequences and performed the phylogenetic analyses; DÓF drafted the manuscript; JBB collected and curated the 1970 museum samples; PPK collected and forwarded preserved bodies of captive specimens; TC, JYM and CH engaged in extensive fieldwork surveys and provided biopsies of extant populations. All authors read and approved the final manuscript.

## Supplementary Material

Additional file 1 Comparative views of *Partula clara incrassa *specimens sampled from Tiapa Valley (Tahiti) by T. Coote in 2007 and by H. E. Crampton during his 1906–1909 expeditions. Click here for file

Additional file 2 Field photograph of suspected *Samoana diaphana *live wild specimen taken by J.-Y. Meyer on Moorea in 2008. Click here for file

Additional file 3 Table showing the taxonomic designation, sampling location, shell voucher specimen catalogue number and GenBank Accession numbers for every partulid mt COI haplotype employed in this study. Click here for file
