# Supplementary material for: Moorean tree snail survival revisited: a multi-island genealogical perspective
Source: BMC Evol Biol. 2009 Aug 18;9:204. doi: 10.1186/1471-2148-9-204 (PMC3087522; doi:10.1186/1471-2148-9-204)
Supplement: Additional file 1 — Comparative views of Partula clara incrassa specimens sampled from Tiapa Valley (Tahiti) by T. Coote in 2007 and by H. E. Crampton during his 1906–1909 expeditions. [file 1471-2148-9-204-S1.doc]

Top plate shows two views of a specimen of *Partula clara incrassa* sampled by Trevor Coote in Tiapa valley, Tahiti, on 08/07/2007 (UMMZ301079).

**
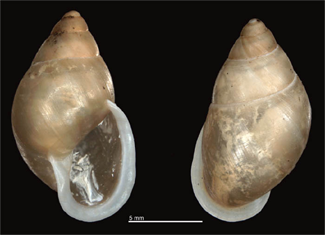
**

Lower plate shows two views of a representative specimen of *Partula clara incrassa*, from Tiapa (Crampton’s Aoua) Valley, Tahiti, collected by H. E. Crampton during his 1906-1909 expeditions. Apart from the faded periostracal lustre, it appears identical to the above specimen. (ANSP276080).

**
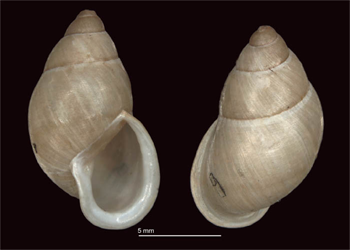
**
