# Supplementary material for: Moorean tree snail survival revisited: a multi-island genealogical perspective
Source: BMC Evol Biol. 2009 Aug 18;9:204. doi: 10.1186/1471-2148-9-204 (PMC3087522; doi:10.1186/1471-2148-9-204)
Supplement: Additional file 2 — Field photograph of suspected Samoana diaphana live wild specimen taken by J.-Y. Meyer on Moorea in 2008. [file 1471-2148-9-204-S2.doc]

A suspected Moorean *Samoana* *diaphana* specimen photographed in the field by Jean-Yves Meyer (March 27^th^ 2008) on a ridge (380 m elevation) above *Col des 3 Cocotiers* under a *Xylosma suaveolens* leaf. Biopsy material is currently unavailable for genotyping purposes but snails in this small relict population had the semi-globose, thin and semi-transparent shell with an obtuse apex that is diagnostic for *S. diaphana* [1]. Pending genetic confirmation of this Moorean population, this species appears to have survived on Moorea as well as on Tahiti.


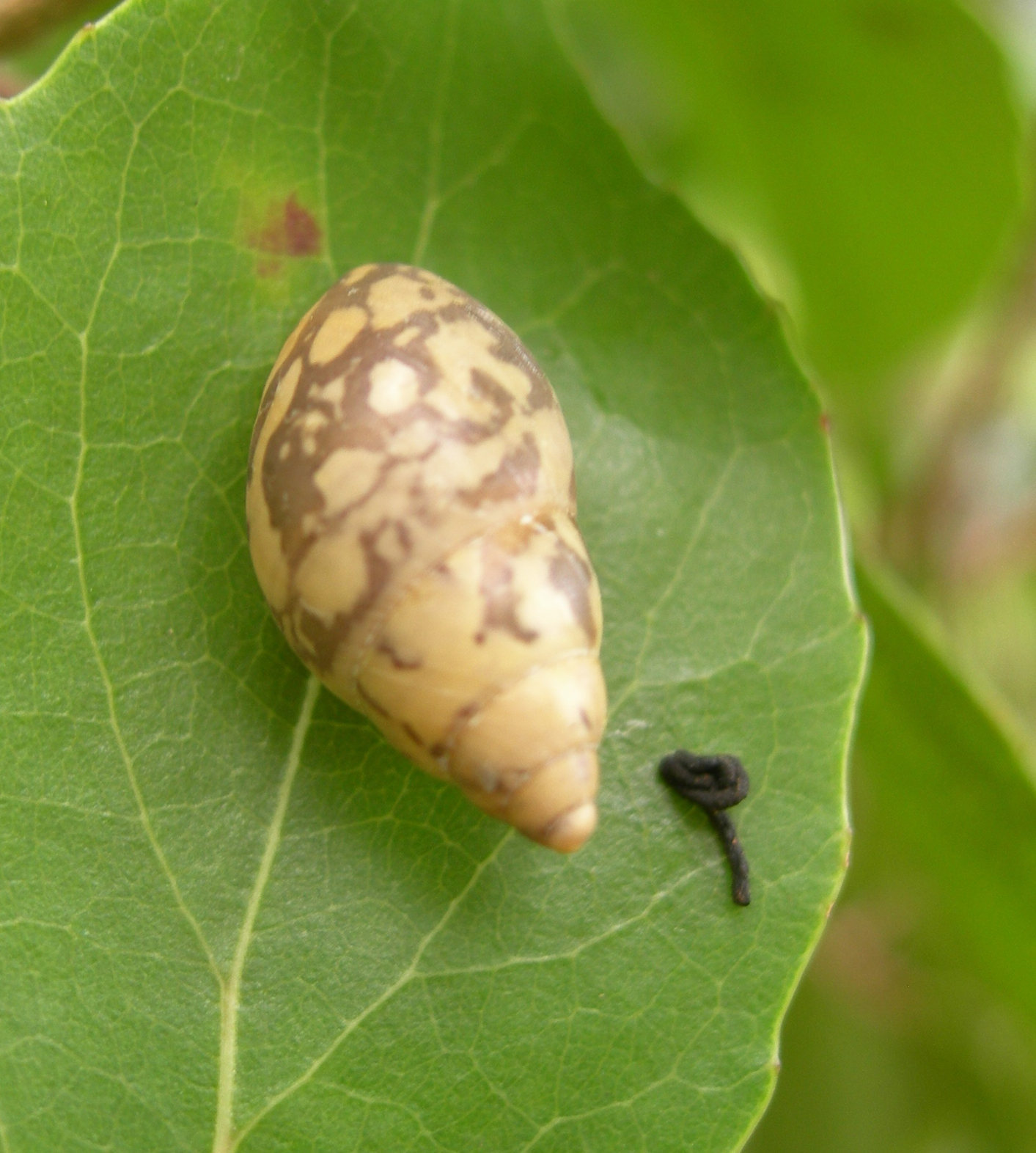


**References**

1. Kondo Y: ***Samoana* of the Society Islands (Pulmonata: Partulidae).** *Malac Rev* 1973, **6:**19-33.
