# Supplementary material for: Moorean tree snail survival revisited: a multi-island genealogical perspective
Source: BMC Evol Biol. 2009 Aug 18;9:204. doi: 10.1186/1471-2148-9-204 (PMC3087522; doi:10.1186/1471-2148-9-204)
Supplement: Additional file 3 — Table showing the taxonomic designation, sampling location, shell voucher specimen catalogue number and GenBank Accession numbers for every partulid mt COI haplotype employed in this study. [file 1471-2148-9-204-S3.doc]

Taxonomic designation, sampling location, shell voucher specimen catalogue number (UMMZ: University of Michigan Museum of Zoology; MNHN: Muséum National d’Histoire Naturelle, Paris; FMNH: Field Museum of Natural History, Chicago) and GenBank Accession numbers (applied for) for every partulid mt COI haplotype employed in the study and incorporated into the phylogenetic trees shown therein. For the Windward Island taxa, Burch’s museum specimens collected in 1970 are indicated in black text, captive zoo specimens are indicated in red text and samples obtained recently (since 2002) from remnant wild populations are indicated in blue text. Sampling details for the Tahitian specimens genotyped in earlier studies are available in the supplementary data of their respective publications [1, 2]. Shell voucher numbers were not available for biopsied wild samples, nor for a small number of 1970 samples that were damaged during the tissue extraction and lyophilization processes.

| Species | Island | Valley Name | Locality number | Collector, Year | # of snails genotyped | Haplotype # | Voucher number | GenBank Accession number (Source) |
| --- | --- | --- | --- | --- | --- | --- | --- | --- |
| *Partula aurantia* Crampton, 1932 | Moorea, Society Is. | Faamaariri V. (300m) | 270 | Burch, 1970 | 3 |  | UMMZ300516, 300517 | EU833005 |
| *P. exigua* Crampton, 1917 | Moorea, Society Is. | Faamaariri V. (150m) | 269 | Burch, 1970 | 2 | 2 | UMMZ300512 | EU833009 |
|  |  | Faamaariri V. (300m) | 270 | Burch, 1970 | 2 | 3 | UMMZ300518 | EU833010 |
|  |  | Puutu V. (400m) | 272 | Burch, 1970 | 2 | 1 | UMMZ300525 | EU833008 |
| *P. mirabilis* Crampton, 1924 | Moorea, Society Is. | Southern slope of Mt. Rotui, Matapoopoo V. (150-200m) | 273 | Burch, 1970 | 1 | 1 | UMMZ300534 | EU833011 |
|  |  |  |  |  | 1 | 2 | UMMZ300531 | EU833012 |
|  |  |  |  |  | 1 | 3 | UMMZ300535 | EU833013 |
|  |  |  |  |  | 1 | 4 | UMMZ300531 | EU833014 |
|  |  |  |  |  | 1 | 5 | UMMZ300528 | EU833015 |
|  |  |  |  |  | 1 | 6 | UMMZ300533 | EU833016 |
|  |  |  |  |  | 11 | 7 | UMMZ300490, 300491, 300526, 300529 | EU833017 |
|  |  | Mouaroa V. (200-300m) | 275 | Burch, 1970 | 4 | 8 | UMMZ300492, 300555 | EU833018 |
|  |  |  |  |  | 1 | 9 | UMMZ300552 | EU833019 |
|  |  | Fareaito V. ? | 11 ? | unknown | 4 | 10 | UMMZ301016 | EU833075 |
| *P. mirabilis propinqua* Crampton, 1932 | Moorea, Society Is. | Matapoopoo V. (150-250m) | 273 | Burch, 1970 | 2 | 1 | Shell voucher not available | EU833061 |
|  |  |  |  |  | 2 | 2 |  | EU833062 |
| *P. mooreana* Hartman, 1880 | Moorea, Society Is. | Hotutea V. northeastern ridge (480m) | 278 | Burch, 1970 | 1 | 1 | UMMZ300547 | EU833020 |
|  |  |  |  |  | 1 | 2 | UMMZ300546 | EU833021 |
|  |  | Hotutea V. northeastern ridge (620m) | 279 | Burch, 1970 | 4 | 1 | Shell voucher not available | EU833020 |
|  |  | Maatea V. | 12 | 1985 | 1 | 4 | UMMZ301017 | EU833073 |
|  |  | Atimaha Ridge | 13 | 1982 | 1 | 3 | UMMZ301018 | EU833074 |
| *P. suturalis* Pfeiffer, 1855 | Moorea, Society Is. | Faamaariri V. (300m) | 270 | Burch, 1970 | 2 | 2 | UMMZ300519 | EU833022 |
|  |  | Puutu V. (250m) | 271 | Burch, 1970 | 3 | 3 | UMMZ300521-300523 | EU833023 |
|  |  | Hotutea V. (620-700m) | 279 | Burch, 1970 | 1 | 1 | UMMZ300557 | EU833024 |
|  |  |  |  |  | 1 | 4 |  | EU833025 |
|  |  |  |  |  | 1 | 5 |  | EU833026 |
|  |  |  |  |  | 1 | 6 |  | EU833027 |
|  |  |  |  |  | 1 | 7 |  | EU833028 |
| *P. s. dendroica* Pfeiffer, 1853 | Moorea, Society Is. | Matapoopoo V. (400m) | 274 | Burch, 1970 | 3 |  | UMMZ300537 | EU833007 |
| *P. s. strigosa* Pfeiffer, 1856 | Moorea, Society Is. | Hotutea V. (400-480m) | 276 | Burch, 1970 | 2 | 1 | UMMZ300556 | EU833029 |
|  |  |  |  |  | 1 | 2 |  | EU833030 |
|  |  |  |  |  | 1 | 7 |  | EU833031 |
|  |  | Hotutea V. (480-620m) | 278 | Burch, 1970 | 1 | 3 | UMMZ300543 | EU833032 |
|  |  | Hotutea V. (620m) | 279 | Burch, 1970 | 1 | 3 | UMMZ300558 | EU833032 |
|  |  |  |  |  | 2 | 4 |  | EU833033 |
|  |  | Maatea V. | 12 | 1980 | 1 | 5 | UMMZ301020 | EU833079 |
|  |  |  |  | 1985 | 1 | 6 | UMMZ301019 | EU833080 |
| *P. s. vexillum* Pease, 1866 | Moorea, Society Is. | Faatoai V. northern slope of Mt. Tautuapae (350m) | 261 | Burch, 1970 | 7 | 9 | UMMZ300487 | EU833034 |
|  |  | Paparoa V. (100m) | 267 | Burch, 1970 | 2 | 2 | UMMZ300494 | EU833035 |
|  |  |  |  |  | 1 | 3 | UMMZ300501 | EU833036 |
|  |  | Paparoa V. (350m) | 268 | Burch, 1970 | 1 | 2 | UMMZ300504, 300505, 300507, 300509 | EU833035 |
|  |  |  |  |  | 1 | 5 |  | EU833037 |
|  |  |  |  |  | 1 | 6 |  | EU833038 |
|  |  |  |  |  | 1 | 7 |  | EU833039 |
|  |  |  |  |  | 1 | 8 |  | EU833040 |
|  |  | Mouaroa V. (200-300m) | 275 | Burch, 1970 | 4 | 4 | UMMZ300538, 300539, 300554 | EU833041 |
|  |  | Hotutea V. (400-480m) | 276 | Burch, 1970 | 1 | 4 | UMMZ300559 | EU833042 |
|  |  | Fareaito V. | 11 | 1982 | 1 | 1 | UMMZ301021 | EU833077 |
|  |  | Vaianai V. | 14 | 1985 | 1 | 10 | UMMZ301022 | EU833078 |
| *P. taeniata* Mörch, 1850 | Moorea, Society Is. | Southern slope of Mt. Rotui, Matapoopoo V. (150-250m) | 273 | Burch, 1970 | 1 | 2 | UMMZ300527,  300532 | EU833043 |
|  |  |  |  |  | 1 | 3 |  | EU833044 |
|  |  |  |  |  | 1 | 7 |  | EU833045 |
|  |  | Maatea V. (180m) | 1 | Coote, 2005, 2006 | 2 | 1 | biopsy | EU833085 |
|  |  | Haumi V. | 2 | Coote, 2005 | 1 | 1 | biopsy | EU833064 |
|  |  | Moruu V. | 3 | Coote, 2006 | 1 | 8 | biopsy | EU833065 |
|  |  | Morioahu V. (243m) | 4 | Coote, 2007 | 1 | 5 | biopsy | EU833088 |
|  |  | Opunohu Bay | 5 | Hickman, 2006 | 5 | 6 | biopsy | EU833071 |
|  |  | Mt. Tohiea (1150m) 17°33’04S 149°49’18W | 6 | Meyer, 2002 | 1 | 4 | biopsy | EU833084 |
| *P. t. elongata* Pease, 1866 | Moorea Society Is. | Paparoa V. (100m) | 267 | Burch, 1970 | 1 | 1 | UMMZ300496, 300498, 300499, 300500, 300502, 300503 | EU833046 |
|  |  |  |  |  | 1 | 5 |  | EU833047 |
|  |  |  |  |  | 1 | 6 |  | EU833048 |
|  |  |  |  |  | 1 | 7 |  | EU833049 |
|  |  |  |  |  | 1 | 8 |  | EU833050 |
|  |  |  |  |  | 1 | 9 |  | EU833051 |
|  |  |  |  |  | 1 | 10 |  | EU833052 |
|  |  | Paparoa V. (300m) | 268 | Burch, 1970 | 1 | 11 | UMMZ300506, 300508 | EU833053 |
|  |  |  |  |  | 1 | 12 |  | EU833054 |
|  |  |  |  |  | 1 | 13 |  | EU833055 |
|  |  | Mouaroa V. (200-300m) | 275 | Burch, 1970 | 1 | 2 | UMMZ300488, 300540, 300542, 300551, 300553 | EU833056 |
|  |  |  |  |  | 4 | 3 |  | EU833057 |
|  |  |  |  |  | 1 | 4 |  | EU833058 |
|  |  |  |  |  | 1 | 14 |  | EU833059 |
| *P. t. nucleola* “Pease” Schmeltz, 1874 | Moorea, Society Is. | Faatoai V. | 261 | Burch, 1970 | 8 | 2 | UMMZ300489 | EU833060 |
|  |  | Faatoai V. | 15 | 1981 | 2 | 1 | UMMZ301023 | EU833081 |
| *P. t. simulans* Pease, 1866 | Moorea, Society Is. | Hotutea V. (400-480m) | 276 | Burch, 1970 | 2 | 2 | UMMZ300544 | EU833063 |
|  |  | Haapiti V. | 16 | 1986 | 2 | 1 | UMMZ301024, 301025 | EU833076 |
|  |  | Mt. Tohiea (1150m) 17°33’04S 149°49’18W | 6 | Meyer, 2006 | 1 | 3 | biopsy | EU833087 |
| *P. tohiveana* Crampton, 1924 | Moorea, Society Is. | Fareaito V. | 11 | 1982 | 1 | 1 | UMMZ301026 | EU833082 |
|  |  |  |  |  | 1 | 2 | UMMZ301027 | EU833083 |
| *P. clara* Pease, 1864 | Tahiti, Society Is. | Tiapa V. (140m) |  | Coote, 2006 | 4 |  | biopsy | EU833072 |
| Tahiti Clade 1  (*P. affinis*/ *otaheitana*) | Tahiti, Society Is. | Punaruu R. V. | 94 | Burch, 1970 | 2 | 16 | UMMZ300359 | EF062929 (Lee *et al*. 2007a) |
|  |  | Faurahi R. V. | 117 | Burch, 1970 | 2 | 15 | UMMZ300378 | EF062970 (Lee *et al*. 2007a) |
|  |  | Moaroa R. V. | 120 | Burch, 1970 | 1 | 116 | UMMZ300386 | EU833006 |
|  |  | Taharuu R. V. | 125 | Burch, 1970 | 4 |  | UMMZ300391 | EF062973 (Lee *et al*. 2007a) |
|  |  | Tirahi R. V. | 138 | Burch, 1970 | 2 | 12 | UMMZ300406, 300408 | EF062928 (Lee *et al*. 2007a) |
|  |  | Vaitepiha V. | 142 | Burch, 1970 | 1 | 1 | UMMZ300411 | EF062875 (Lee *et al*. 2007a) |
|  |  | Vaitehoro R. V | 152 | Burch, 1970 | 1 | 18 | UMMZ300417 | EF062873 (Lee *et al*. 2007a) |
|  |  |  |  |  | 2 | 19 |  | EF062872 (Lee *et al*. 2007a) |
|  |  | Taravao Plateau | 157 | Burch, 1970 | 2 | 17 | UMMZ300440 | EF062950 (Lee *et al*. 2007a) |
|  |  | Ahaavini V. | 159 | Burch, 1970 | 1 | 21 | UMMZ300419,  300421-300424 | EF062948 (Lee *et al*. 2007a) |
|  |  |  | 161 | Burch, 1970 | 1 | 23 | UMMZ300441, 300442 | EF062874 (Lee *et al*. 2007a) |
|  |  | Vaiarava R. V. | 162 | Burch, 1970 | 1 | 2 | UMMZ300443 | EF062979 (Lee *et al*. 2007a) |
|  |  | Onoheha V. | 186 | Burch, 1970 | 1 | 3 | UMMZ300425 | EF062946 (Lee *et al*. 2007a) |
|  |  | Tetiairiroa V | 192 | Burch, 1970 | 2 | 5 | UMMZ 300467, 300470 | EF062870 (Lee *et al*. 2007a) |
|  |  |  |  |  | 1 | 6 | UMMZ300468 | EF062944 (Lee *et al*. 2007a) |
|  |  | Tetiairiroa V. | 192 | Burch, 1970 | 1 | 10 | UMMZ300469 | EF062943 (Lee *et al*. 2007a) |
|  |  |  |  |  | 1 | 11 | UMMZ300468 | EF062945 (Lee *et al*. 2007a) |
|  |  | Fareteuira R. V. | 198 | Burch, 1970 | 3 | 4 | UMMZ300474, 300475 | EF062977 (Lee *et al*. 2007a) |
|  |  |  |  |  | 1 | 8 | UMMZ300476 | EF062871 (Lee *et al*. 2007a) |
|  |  |  |  |  | 1 | 9 | UMMZ300474 | EF062978 (Lee *et al*. 2007a) |
|  |  | Tuauru R. V. | 211 | Burch, 1970 | 1 | 7 | UMMZ300482 | EF062869 (Lee *et al*. 2007a) |
|  |  | Taravao Plateau | 243 | Burch, 1970 | 1 | 20 | UMMZ300434 | EF062951 (Lee *et al*. 2007a) |
|  |  | Te Pari District | 3 | 1995 | 2 | 13 | UMMZ300560, 300561 | EF062882 (Lee *et al*. 2007a) |
|  |  |  |  |  | 3 | 14 | UMMZ300561 | EF062883 (Lee *et al*. 2007a) |
|  |  | Mt. Atara, Taravao Plateau (1050m) | 10 | Coote, 2005 | 1 | 22 | biopsy | EF062952 (Lee *et al*. 2007a) |
|  |  |  |  |  | 1 | 24 | biopsy | EF062931 (Lee *et al*. 2007a) |
|  |  |  |  |  | 1 | 25 | biopsy | EF062985 (Lee *et al*. 2007a) |
|  |  |  |  |  | 1 | 26 | biopsy | EF062984 (Lee *et al*. 2007a) |
| Tahiti Clade 2  (*P. filosa*/*otaheitana*) | Tahiti, Society Is. | Punaruu R. V | 90 | Burch, 1970 | 3 | 47 | UMMZ300357 | EF062937 (Lee *et al*. 2007a) |
|  |  |  | 94 | Burch, 1970 | 1 | 46 | UMMZ300358 | EF062983 (Lee *et al*. 2007a) |
|  |  | Fautaua R. V. | 144 | Burch, 1970 | 1 | 35 | UMMZ300414 | EF062941 (Lee *et al*. 2007a) |
|  |  |  |  |  | 2 | 37 | UMMZ300414 | EF062940 (Lee *et al*. 2007a) |
|  |  |  | 221 | Burch, 1970 | 2 | 34 | UMMZ300484 | EF062942 (Lee *et al*. 2007a) |
|  |  |  |  |  | 1 | 36 | UMMZ300485 | EF062953 (Lee *et al*. 2007a) |
|  |  | Pirae V. | 200 | Burch, 1970 | 4 | 29 | UMMZ300428, 300429 | EF062905 (Lee *et al*. 2007a) |
|  |  |  |  |  | 1 | 38 | UMMZ300479 | EF062907 (Lee *et al*. 2007a) |
|  |  |  |  |  | 3 | 39 | UMMZ300477 | EF062976 (Lee *et al*. 2007a) |
|  |  |  |  |  | 1 | 40 | UMMZ300479 | EF062906 (Lee *et al*. 2007a) |
|  |  | Tipaerui V. | 217 | Burch, 1970 | 2 | 30 | UMMZ300430 | EF062938 (Lee *et al*. 2007a) |
|  |  |  |  |  | 1 | 31 | UMMZ300431 | EF062939 (Lee *et al*. 2007a) |
|  |  | Mt. Marau, Belevedere | 5 | 1995 | 1 | 42 | UMMZ300567 | EF062932 (Lee *et al*. 2007a) |
|  |  | Mt. Aorai, Fare Mato (1300m) | 1 | Coote, 2006 | 1 | 32 | biopsy | EF462396 (Lee *et al*. 2007a) |
|  |  | Mt. Aorai, Fare Mato (1400m) | 1 | Coote, 2006 | 1 | 33 | biopsy | EF462395 (Lee *et al*. 2007a) |
|  |  | Mt. Aorai, Fare Mato (1402m) | 1 | Coote, 2007 | 1 | 115 | biopsy | EU833089 |
|  |  | Mt. Marau, Site 1 | 16 | Coote, 2005 | 2 | 44 | biopsy | EF062989 (Lee *et al*. 2007a) |
|  |  |  |  |  | 1 | 45 | biopsy | EF062990 (Lee *et al*. 2007a) |
|  |  | Mt. Marau, Belvedere | 17 | Coote, 2005 | 1 | 41 | biopsy | EF062986 (Lee *et al*. 2007a) |
|  |  |  |  |  | 1 | 43 | biopsy | EF062987 (Lee *et al*. 2007a) |
|  |  | Mt. Marau (1246m) |  | Coote, 2007 | 1 | 117 | biopsy | EU833090 |
|  |  |  |  |  | 1 | 118 | biopsy | EU833091 |
|  |  | Mt. Marau (1391m) |  | Coote, 2007 | 1 | 119 | biopsy | EU833092 |
| Tahiti Clade 3  (*P. affinis*/*nodosa*/ *otaheitana*/*producta*) | Tahiti, Society Is. | Punaruu R. V. | 91 | Burch, 1970 | 3 | 67 | shell voucher unavailable | EF062925 (Lee *et al*. 2007a) |
|  |  | Orofero V. | 99 | Burch, 1970 | 1 | 75 | UMMZ300361 | EF062982 (Lee *et al*. 2007a) |
|  |  | Afeu R. V. | 107 | Burch, 1970 | 2 | 61 | UMMZ300363 | EF062877 (Lee *et al*. 2007a) |
|  |  | Temarua V. | 108 | Burch, 1970 | 3 | 49 | UMMZ300368, 300369 | EF062955 (Lee *et al*. 2007a) |
|  |  |  | 110 | Burch, 1970 | 1 | 50 | UMMZ300372 | EF062957 (Lee *et al*. 2007a) |
|  |  |  |  |  | 1 | 51 | UMMZ300372 | EF062956 (Lee *et al*. 2007a) |
|  |  |  |  |  | 1 | 70 | UMMZ300372 | EF062958 (Lee *et al*. 2007a) |
|  |  |  | 113 | Burch, 1970 | 1 | 52 | UMMZ300376 | EF062959 (Lee *et al*. 2007a) |
|  |  |  |  |  | 1 | 62 | UMMZ300377 | EF062961 (Lee *et al*. 2007a) |
|  |  | Faurahi R. V. | 117 | Burch, 1970 | 1 | 58 | UMMZ300378 | EF062969 (Lee *et al*. 2007a) |
|  |  |  |  |  | 3 | 69 | UMMZ300379 | EF062975 (Lee *et al*. 2007a) |
|  |  | Moaroa R. V. | 120 | Burch, 1970 | 1 | 53 | UMMZ300385, 300387 | EF062971 (Lee *et al*. 2007a) |
|  |  |  |  |  | 1 | 54 | UMMZ 300385, 300387 | EF062972 (Lee *et al*. 2007a) |
|  |  | Papeiti R. | 123 | Burch, 1970 | 1 | 48 | UMMZ300389, 300390 | EF062954 (Lee *et al*. 2007a) |
|  |  | Taharuu R. V. | 125 | Burch, 1970 | 1 | 55 | UMMZ300391 | EF062974 (Lee *et al*. 2007a) |
|  |  | Toheimahu R. V. | 135 | Burch, 1970 | 1 | 68 | UMMZ300397 | EF062947 (Lee *et al*. 2007a) |
|  |  | Tahiria R. V. | 169 | Burch, 1970 | 1 | 57 | UMMZ300451 | EF062966 (Lee *et al*. 2007a) |
|  |  |  |  |  | 1 | 60 | UMMZ300450 | EF062965 (Lee *et al*. 2007a) |
|  |  |  | 170 | Burch, 1970 | 1 | 56 | UMMZ300455 | EF062968 (Lee *et al*. 2007a) |
|  |  |  |  |  | 1 | 59 | UMMZ300455 | EF062967 (Lee *et al*. 2007a) |
|  |  | Tereia R. V. | 178 | Burch, 1970 | 1 | 71 | UMMZ300464 | EF062881 (Lee *et al*. 2007a) |
|  |  |  |  |  | 1 | 72 | UMMZ300461 | EF062981 (Lee *et al*. 2007a) |
|  |  |  |  |  | 2 | 73 | UMMZ300462, 300463 | EF062980 (Lee *et al*. 2007a) |
|  |  |  | 180 | Burch, 1970 | 1 | 73 | UMMZ300465 | EF062962 (Lee *et al*. 2007a) |
|  |  | Paihau R. V. | 224 | Burch, 1970 | 2 | 74 | shell voucher unavailable | EF062936 (Lee *et al*. 2007a) |
|  |  | Mt. Marau, Belevedere | 5 | 1995 | 1 | 63 | UMMZ300570 | EF062935 (Lee *et al*. 2007a) |
|  |  | Papehue R. V. | 44 | 1984 | 3 | 65 | UMMZ300572 | EF062926 (Lee *et al*. 2007a) |
|  |  |  |  |  | 3 | 66 | UMMZ300565, UMMZ300566 | EF062927 (Lee *et al*. 2007a) |
|  |  | Mt. Marau, Site 1 | 16 | Coote, 2005 | 1 | 64 | biopsy | EF062988 (Lee *et al*. 2007a) |
| Tahiti Clade 4  (*P. affinis*/*otaheitana*) | Tahiti, Society Is. | Afeu R. V. | 106 | Burch, 1970 | 1 | 83 | UMMZ300362 | EF062963 (Lee *et al*. 2007a) |
|  |  |  | 107 | Burch, 1970 | 1 | 76 | UMMZ300365, 300366 | EF062964 (Lee *et al*. 2007a) |
|  |  | Temarua V. | 113 | Burch, 1970 | 1 | 77 | UMMZ300376 | EF062960 (Lee *et al*. 2007a) |
|  |  | Ahaavini V. | 159 | Burch, 1970 | 1 | 82 | UMMZ300419,  300421-300424 | EF062949 (Lee *et al*. 2007a) |
|  |  | Tiitauiri R. V | 174 | Burch, 1970 | 1 | 80 | UMMZ300452 | EF062878 (Lee *et al*. 2007a) |
|  |  |  | 176 | Burch, 1970 | 1 | 79 | UMMZ300460 | EF062880 (Lee *et al*. 2007a) |
|  |  |  |  |  | 2 | 81 | UMMZ300457 | EF062879 (Lee *et al*. 2007a) |
|  |  | Mt. Marau, Belevedere | 5 | 1995 | 1 | 78 | UMMZ300567 | EF062933 (Lee *et al*. 2007a) |
| Tahiti Clade 5  (*P. clara*/*hyalina*) | Tahiti, Society Is. | Orofero V. | 99 | Burch, 1970 | 3 | 112 | UMMZ300360 | EU026162 (Lee *et al*. 2007b) |
|  |  | Afeu R. V. | 107 | Burch, 1970 | 4 | 96 | UMMZ300367 | EF062914 (Lee *et al*. 2007a) |
|  |  | Moaroa R. V. | 120 | Burch, 1970 | 26 | 96 | UMMZ300382, 300383, 300386, 300388 | EF062892 (Lee *et al*. 2007a) |
|  |  | Taharuu R. V. | 125 | Burch, 1970 | 2 | 87 | UMMZ300393 | EF062893 (Lee *et al*. 2007a) |
|  |  | Mapuaura R. V. | 131 | Burch, 1970 | 3 | 97 | UMMZ300395 | EF062912 (Lee *et al*. 2007a) |
|  |  | Toheimahu R. V | 135 | Burch, 1970 | 1 | 96 | UMMZ300396, 300398 | EF062884 (Lee *et al*. 2007a) |
|  |  | Tirahi R. V. | 136 | Burch, 1970 | 3 | 94 | UMMZ300401 | EF062887 (Lee *et al*. 2007a) |
|  |  |  | 138 | Burch, 1970 | 2 | 95 | UMMZ300407 | EF062913 (Lee *et al*. 2007a) |
|  |  | Vaitepiha V. | 142 | Burch, 1970 | 4 | 107 | UMMZ300412 | EF062886 (Lee *et al*. 2007a) |
|  |  | Fautaua R. V. | 144 | Burch, 1970 | 11 | 107 | UMMZ300413 | EF062908 (Lee *et al*. 2007a) |
|  |  |  | 220 | Burch, 1970 | 8 | 107 | shell voucher unavailable | EF062908 (Lee *et al*. 2007a) |
|  |  |  | 222 | Burch, 1970 | 7 | 107 | shell voucher unavailable | EF062908 (Lee *et al*. 2007a) |
|  |  |  |  |  | 2 | 94 | shell voucher unavailable | EF062909 (Lee *et al*. 2007a) |
|  |  | Vaitehoro R. V. | 152 | Burch, 1970 | 2 | 97 | UMMZ300435 | EF062885 (Lee *et al*. 2007a) |
|  |  | Vairaharaha R. V. | 165 | Burch, 1970 | 6 | 86 | UMMZ300426 | EF062891 (Lee *et al*. 2007a) |
|  |  | Tahiria R. V. | 169 | Burch, 1970 | 1 | 85 | UMMZ300454 | EF062889 (Lee *et al*. 2007a) |
|  |  |  |  |  | 2 | 88 | UMMZ300456 | EF062889 (Lee *et al*. 2007a) |
|  |  |  | 170 | Burch, 1970 | 1 | 85 | UMMZ300453 | EF062888 (Lee *et al*. 2007a) |
|  |  |  |  |  | 1 | 89 | UMMZ300456 | EF062890 (Lee *et al*. 2007a) |
|  |  | Tetiairiroa V. | 192 | Burch, 1970 | 1 | 102 | UMMZ300471 | EF062911 (Lee *et al*. 2007a) |
|  |  | Tuauru R. V | 211 | Burch, 1970 | 4 | 106 | UMMZ300483 | EF062910 (Lee *et al*. 2007a) |
|  |  | Tipaerui V. | 217 | Burch, 1970 | 5 | 105 | shell voucher unavailable | EF062915 (Lee *et al*. 2007a) |
|  |  | Paihau R. V. | 224 | Burch, 1970 | 4 | 104 | shell voucher unavailable | EF062894 (Lee *et al*. 2007a) |
|  |  | Papenoo-Maroto V. | 1 | 1997 | 2 | 90 | UMMZ300563 | EF062901 (Lee *et al*. 2007a) |
|  |  |  |  |  | 1 | 91 | UMMZ300563 | EF062903 (Lee *et al*. 2007a) |
|  |  |  |  |  | 1 | 92 | UMMZ300563 | EF062902 (Lee *et al*. 2007a) |
|  |  | Tahaute V. | 2 | 1996 | 4 | 93 | UMMZ300564 | EF062924 (Lee *et al*. 2007a) |
|  |  | Te Pari District | 3 | 1995 | 4 | 96 | UMMZ300562 | EF062904 (Lee *et al*. 2007a) |
|  |  | Ahonu R. V. | 2 | Coote, 2006 | 1 | 106 | biopsy | EF062916 (Lee *et al*. 2007a) |
|  |  | Faarapa V. | 3 | Coote, 2005 | 1 | 105 | biopsy | EF062895 (Lee *et al*. 2007a) |
|  |  | Haapupuni V. | 4 | Coote, 2005 | 2 | 98 | biopsy | EF062917 (Lee *et al*. 2007a) |
|  |  | Onoheha-Tefaaiti V. | 5 | Coote, 2004 | 1 | 105 | biopsy | EF062896 (Lee *et al*. 2007a) |
|  |  |  | 6 | Coote, 2004 | 1 | 103 | biopsy | EF062918 (Lee *et al*. 2007a) |
|  |  | Tahaute V. | 7 | Coote, 2005 | 1 | 103 | biopsy | EF062919 (Lee *et al*. 2007a) |
|  |  | Vaitoare R. V. | 8 | Coote, 2005 | 1 | 99 | biopsy | EF062920 (Lee *et al*. 2007a) |
|  |  | Raroui R. V. | 9 | Coote, 2005 | 1 | 94 | biopsy | EF062921 (Lee *et al*. 2007a) |
|  |  |  |  |  | 1 | 97 | biopsy | EF062922 (Lee *et al*. 2007a) |
|  |  | Vaipahi R. V. | 11 | Coote, 2004 | 1 | 96 | biopsy | EF062897 (Lee *et al*. 2007a) |
|  |  | Taapua V. | 12 | Coote, 2005 | 1 | 97 | biopsy | EF062898 (Lee *et al*. 2007a) |
|  |  | Papehue R. V. (175m) | 13 | Coote, 2006 | 1 | 101 | biopsy | EF062899 (Lee *et al*. 2007a) |
|  |  | Maruapo V. (120m) | 14 | Coote, 2005 | 1 | 100 | biopsy | EF062900 (Lee *et al*. 2007a) |
|  |  | Matatia V. (120m) | 15 | Coote, 2005 | 1 | 93 | biopsy | EF062923 (Lee *et al*. 2007a) |
|  |  | Tihiute R. V. (120m) | 18 | Coote, 2006 | 1 | 111 | biopsy | EU026163 (Lee *et al*. 2007b) |
|  |  | Ahaavini V. (90m) |  | Coote, 2007 | 1 | 94 | biopsy | EU833093 |
|  |  | Taapeha V. (<30m) |  | Coote, 2007 | 1 | 96 | biopsy | EU833094 |
|  |  | Fautaua V. (490m) |  | Coote, 2007 | 1 | 113 | biopsy | EU833095 |
|  |  | Vaipoe V. (272m) |  | Coote, 2007 | 1 | 114 | biopsy | EU833096 |
|  | Raivavae, Ausral Is. | 23°85709S, 147°61591W (20m) |  | Fontaine & Gargominy, 2002 | 2 | 107 | MNHNRv39 | EU026171 (Lee *et al*. 2007b) |
|  |  | 23°87467S, 147°68989W (90m) |  | Fontaine & Gargominy, 2002 | 1 | 107 | MNHNRv71 | EU026172 (Lee *et al*. 2007b) |
|  | Rimatara, Austral Is. | 22°63868S, 152°80645W (10m) |  | Fontaine & Gargominy, 2004 | 4 | 97 | MNHNRm31 | EU026168 (Lee *et al*. 2007b) |
|  |  |  |  |  | 1 | 108 |  | EU026169 (Lee *et al*. 2007b) |
|  | Rurutu, Austral Is. | Falises de Matonaa (<10m) |  | Coote, 2005 | 1 | 90 | UMMZ300610 | EU026164 (Lee *et al*. 2007b) |
|  |  | Mato Arei, 22°45687S, 151°32423W (10m) |  | Fontaine & Gargominy, 2003 | 1 | 109 | MNHNRr06 | EU026166 (Lee *et al*. 2007b) |
|  |  | S. Paparai, 22°50772S, 151°33412W (60m) |  | Fontaine & Gargominy, 2003 | 1 | 109 | MNHNRr36 | EU026167 (Lee *et al*. 2007b) |
|  |  | Pte Arei, Peva (<20m) |  | Coote, 2005 | 1 | 110 | UMMZ300611 | EU026165 (Lee *et al*. 2007b) |
|  | Tubuai, Austral Is. | 23°38198S, 149°52343W (2m) |  | Fontaine & Gargominy, 2003 | 1 | 97 | MNHNTb11 | EU026170 (Lee *et al*. 2007b) |
|  | Mangaia, Cook Is. |  |  | McCormack, 2006 | 6 | 97 | UMMZ300613-300617 | EU026175-026179  (Lee *et al*. 2007b) |
|  | Mauke, Cook Is |  |  | Coote, 2007 | 4 | 107 | UMMZ300612 | EU026173, 026174  (Lee *et al*. 2007b) |
| Tahiti Haplotype 28 | Tahiti, Society Is. | Tirahi R. V. | 139 | Burch, 1970 | 2 | 28 | UMMZ300409 | EF062876 (Lee *et al*. 2007a) |
| Tahiti Haplotype 84 | Tahiti, Society Is. | Mt. Aorai, Fare Mato | 1 | Coote, 2005 | 1 | 84 | biopsy | EF062930 (Lee *et al*. 2007a) |
| Tahiti Haplotype 120 | Tahiti, Society Is. | Pointe Terurua |  | Coote, 2007 | 1 | 120 | biopsy | EU833097 |
| *Samoana attenuata* (Pease, 1864) | Moorea, Society Is. | Mt. Tohiea Belvedere *Partula* reserve | 7 | Hickman, 2006 | 1 | 5 | biopsy | EU833086 |
|  | Tahiti, Society Is. | Tiitauiri R. V. | 175 | Burch, 1970 | 1 | 3 | UMMZ300619 | EU026193 (Lee *et al*. 2008) |
|  |  | Fareteuira R. V. | 198 | Burch, 1970 | 1 | 2 | UMMZ300427 | EU832999 |
|  |  | Haapupuni V. (115m) |  | Coote, 2005 | 1 | 1 | biopsy | EU833066 |
|  | Raiatea, Society Is. | Tefatua, Mt. Toomaru |  | Meyer, 2006 | 1 | 4 | UMMZ300620 | EU026194 (Lee *et al*. 2008) |
| *S. burchi* Kondo, 1973 | Tahiti, Society Is. | Taravao Plateau | 243 | Burch, 1970 | 2 | 1 | UMMZ300433 | EU833000 |
|  |  |  |  |  | 4 | 2 |  | EU833001 |
|  |  |  | 157 | Burch, 1970 | 2 | 2 | UMMZ300432 | EU833001 |
|  |  | Mt. Atara (1000m) |  | Coote, 2005 | 1 | 3 | biopsy | EU833070 |
|  |  | Mt. Aorai |  | Coote, 2005 | 1 | 4 | biopsy | EU833067 |
| *S. diaphana* Crampton & Cooke, 1953 | Moorea, Society Is. | Hotutea V. | 279 | Burch, 1970 | 2 | 1 | UMMZ300548 | EU833002 |
|  |  |  |  |  | 1 | 2 |  | EU833003 |
|  |  |  |  |  | 1 | 3 |  | EU833004 |
|  | Tahiti, Society Is. | Mt. Aorai, (1300m) |  | Coote, 2006 | 1 | 4 | biopsy | EU833098 |
|  |  | Mt. Aorai (1157m) |  | Coote, 2007 | 1 |  | biopsy | EU833098 |
|  |  | Mt. Pihaaiateta |  | Halland, 2004 | 1 | 5 | biopsy | EU833099 |
| *Eua expansa* (Pease, 1872) | Savaii, Samoa | Savaii |  | Price, 1965 | 1 |  | FMNH152561 | EU833069 |
| *E. globosa* Pilsbry & Cooke, 1934 | Eua, Tonga |  |  | Burch, 1970 | 2 |  | UMMZ300563 | EU832996 |
| *E. montana* (Cooke & Crampton, 1930) | Upolu, Samoa | Upolu |  | Solem & Price, 1965 | 1 |  | FMNH152750 | EU833068 |
| *E. zebrina* (Gould, 1946) | Samoa |  |  |  | 1 | 1 |  | AY148566 (acquired from GenBank) |
|  | Tutuila, American Samoa |  |  | Burch, 1970 | 1 | 2 | UMMZ300562 | EU832997 |
|  |  |  |  |  | 1 | 3 |  | EU832998 |

**References Cited**

1. Lee T, Burch JB, Jung Y, Coote T, Pearce-Kelly P, Ó Foighil D: **Tahitian tree snail mitochondrial clades survived recent mass-extirpation.** *Curr Biol* 2007,**17:**R502-R503.

2. Lee T, Burch JB, Coote T, Fontaine B, Gargominy O, Pearce-Kelly P, Ó Foighil D: **Prehistoric inter-archipelago trading of Polynesian tree snails leaves a conservation legacy.** *Proc R Soc Lond B* 2007, **272:**2907-2914.
